# Psychometric properties of an Arabic translation of the short 9-item drive for muscularity scale (DMS-9)

**DOI:** 10.1186/s12888-023-05179-9

**Published:** 2023-09-19

**Authors:** Feten Fekih-Romdhane, Diana Malaeb, Mariam Dabbous, Rabih Hallit, Sahar Obeid, Souheil Hallit

**Affiliations:** 1 The Tunisian Center of Early Intervention in Psychosis, Department of Psychiatry “Ibn Omrane”, Razi hospital, Manouba, 2010 Tunisia; 2 Faculty of Medicine of Tunis, Tunis El Manar University, Tunis, Tunisia; 3 College of Pharmacy, Medical Gulf University, Ajman, United Arab Emirates; 4 School of Pharmacy, Lebanese International University, Beirut, Lebanon; 5 School of Medicine and Medical Sciences, Holy Spirit University of Kaslik, P.O. Box 446, Jounieh, Lebanon; 6 Department of Infectious Disease, Bellevue Medical Center, Mansourieh, Lebanon; 7 Department of Infectious Disease, Notre Dame des Secours University Hospital, Postal code 3, Byblos, Lebanon; 8 Social and Education Sciences Department, School of Arts and Sciences, Lebanese American University, Jbeil, Lebanon; 9 Psychology Department, College of Humanities, Effat University, Jeddah, 21478 Saudi Arabia; 10 Applied Science Research Center, Applied Science Private University, Amman, Jordan; 11 Research Department, Psychiatric Hospital of the Cross, Jal Eddib, Lebanon

**Keywords:** Drive for muscularity, Body image, Arabic, Validation, Psychometric properties

## Abstract

**Background:**

After the original 15-item Drive for Muscularity Scale developed by McCreary et al. in 2004, a more theoretically based scale that replicates the original DMS subscales with a better conceptual clarity and a shorter number of items, i.e., the DMS-9, has recently been developed by Chaba et al. in 2018. We sought to contribute to the literature especially under the Arab context, by investigating the psychometric properties of an Arabic translation of the DMS-9 in a sample of Arabic-speaking Lebanese university students of both genders.

**Methods:**

University students (N = 402; 55.2% females) from multiple universities in Lebanon were invited to fill the survey in this cross-sectional designed study (December 2022 and January 2023). Our sample was chosen using the snowball technique; a soft copy of the questionnaire was created using google forms software, and an online approach was conceived to proceed with the data collection.

**Results:**

Using an Exploratory Factor Analysis-to- Confirmatory Factor Analysis strategy, we found that the original two-factor model of the DMS proposed in the parent study was adequately replicated in our sample. The two DMS-9 factor scores showed very good McDonald’s omega values (ω > 0.8). Findings also showed that gender invariance was achieved at the configural, metric, and scalar levels. Additionally, drive for muscularity scores correlated in the expected way with other study variables, providing support for the convergent and divergent validity of the Arabic DMS-9. Specifically, we found that greater drive for muscularity attitudes and behaviors significantly correlated with more severe muscle dysmorphic symptoms, inappropriate eating attitudes, muscle bias internalization, and lower body appreciation.

**Conclusion:**

Findings preliminarily suggest that the Arabic DMS-9 is psychometrically sound and suitable tool to assess the drive for muscularity construct among Arabic-speaking community adults. Making the Arabic DMS-9 available will hopefully benefit the scientific community working in Arab settings, promote local and international research in this area, and offer descriptive data on how drive for muscularity may interfere with health indicators in the general Arab population.

## Introduction

Drive for muscularity is defined as a desire to develop a visible muscular physique by both increasing muscle mass and decreasing body fat, in order to achieve a muscular upper body and a narrow waist [1–3]. Striving to attain a muscular ideal body gets affected individuals to highly engage in appearance-related cognitions and various muscularity-building behaviors [4–6]. Men who desire valorized musculatures are at risk to develop multiple psychological and physical health consequences. More specifically, drive for muscularity was found to relate to depression, anxiety, decreased self-esteem, and substance use intentions [5, 7, 8]. Men driven to achieve an idealized muscular body are also more prone to use steroids [7], to report exercise dependence [9], disordered eating [10] and bulimic symptoms [10, 11]. All these negative consequences highlight the crucial necessity for adequate management of drive for muscularity. Drive for muscularity can be successfully managed and treated when properly captured and timely diagnosed [12]. However, there is some evidence to suggest that muscularity-oriented disordered eating and body image in males are still largely misunderstood by clinicians, underdiagnosed and undertreated [13]. These problems remain also underreported by males, partly because of stigma and shame attached to them [14]; which often lead to reluctance to seek help and substantial delays to care [15, 16]. Hence, early screening and assessment for drive for muscularity is of great importance, as it could be the key to detect the problem as early as possible and successfully manage it before symptoms become disabling.

No gold-standard measure of the drive for muscularity exists [17]. Several measures exist to evaluate the drive for muscularity construct, such as the Drive for Muscularity Attitudes Questionnaire (DMAQ; [18]), the Swansea Muscularity Attitudes Questionnaire (SMAQ; [19]), and the Drive for Muscularity Scale (DMS; [20]). In this study, we chose to validate the Arabic version of the DMS, given that it is the most commonly used measure to assess drive for muscularity (70% of studies [21]). The DMS is a self-report measure composed of 15 items that are rated on a 6-point Likert-type scale (from 1 “always” to 6 “never”). The original developers obtained a structure of two factors, each one composed of 7 items (i.e., Muscularity Body Dissatisfaction and Muscularity Behaviors) in a sample of North American men [22]. The Muscularity Body Dissatisfaction subscale reflects one’s “attitudes” toward muscle-oriented body image, while the Muscularity Behaviors subscale reflects engaging in “behaviors” that promote a gain in muscle mass [22]. One item (#10: “I think about taking anabolic steroids”) was found to have very little variability and was omitted from the subscale calculations [22]. Higher total scores are indicative of more pronounced attitudes and behaviors of drive for muscularity. With regard to the DMS factor structure, the same study by McCreary et al. (2004) [22] has shown that, for men, researchers can compute separate attitude and behavioral subscale scores and an overall DMS score. However, for women, only the overall DMS score can be computed.

Since its development, the DMS has been translated in different languages and adapted to different countries and languages, including Spanish [23, 24], Italian [25], German [26], Portuguese [27], Romanian [28], Turkish [29], Persian [30], Lithuanian [31], Malay [32], Brazilian [33], and Chinese [34]. The DMS has also been validated in various populations, including university students men [20, 28], young adult women [35], sexual minority men and women [36, 37], weightlifters [38], and bodybuilders [30]. All these versions provided empirical support to the good psychometric characteristics of the DMS, by showing an adequate internal consistency (Cronbach’s alpha > 0.70) and confirming its original 2-factor structure (attitudes and behaviors) [39]. It is of note, however, that the vast majority of evidence was originated from exclusively men samples [39]. The very limited attempts to validate the measure in women samples (e.g., [22, 40, 41]) failed to support the factor model suggested in the parent version and consistently described in men; thus questioning the factorial validity of the scale and its invariance across gender groups. Other psychometric characteristics have also been supported, including test–retest reliability [3, 26, 31], and good convergent validity as evidenced through significant correlations with other relevant constructs (e.g., body image dissatisfaction [23, 42], self-esteem [1, 20, 31, 32], muscle discrepancy [32], BMI [31, 32], disordered eating attitudes/behaviors [1, 20, 31], and psychological distress [1, 38]).

Although the 15-item DMS has been widely validated and extensively used in diverse research and clinical settings, and the findings that it relates to different relevant constructs (e.g., socio-demographic variables, drive for thinness, drive for leanness) [3], this original version was not theoretically driven [43]. Indeed, despite attempts to conceptualize the scale on two separate dimensions, there is a lack of clarity surrounding this conceptualization. For instance, the Muscularity Attitudes subscale includes items referring to various theoretical constructs, such as self-efficacy (e.g., “I think that I would feel stronger if I gained a little more muscle mass”) or subjective norms/social approval (e.g., “Other people think I work out with weights too often”). To fill these gaps, Chaba et al. [43] sought to establish a more theoretically based scale that replicates the original DMS subscales with a better conceptual clarity and a shorter number of items. To this end, they developed a preliminary version based on both the literature on the drive for muscularity and the first version of the DMS, and investigated its factor structure with principal component analysis in a sample of 114 male athletes [43]. This has led to a nine-item scale that demonstrated good psychometric properties using series of structural hypothetical modelisation in another sample of 129 male athletes [43]. The short 9-item DMS (DMS-9) was therefore shown to be conceptualized on two theoretical factors, Muscularity Body Dissatisfaction and Muscularity Behaviors [43]. Given that this new version of the scale is theoretically sounder, it has the potential to offer a clearer approach to understanding the drive for muscularity construct. In addition, due to its shortness, the DMS-9 allows for easier use, shorter administration time, less respondents’ burden and lower cost compared to the original form.

### Rationale of the present study

To date, no Arabic validation of the DMS exists to the best of our knowledge. Although research on disordered eating and body image disturbances has been widely developed in the Western world, the generalized globalization and westernization contributed to a rise in prevalence rates of these manifestations in people from non-Western cultures even exceeding those seen in Western people [44, 45]. Research has, for example, documented a growing prevalence of maladaptive eating-related attitudes and behaviors in the Arab world [46, 47]. Despite this evidence, little attention has been devoted so far to this topic in Arab countries; which is partly due to a lack of sensitive measures to detect manifestations of muscularity-oriented nature in Arabic-speaking populations [48]. Available instruments in the Arabic language are rather thinness-focused, such as the Eating Attitude Test [49, 50], the Eating disorder examination questionnaire [51, 52], the Inflexible Eating Questionnaire (IEQ) [53], the Nine Item Avoidant/Restrictive Food Intake Disorder Screen (NIAS) [54], the Intuitive Eating Scale [55], and the Eating Disorder Inventory [56, 57]. The only muscularity-specific measure that has recently been validated in Arabic is the Muscle Dysmorphic Disorder Inventory [58]. In addition, studies on body dissatisfaction involving Arab men samples are scarce, with a great majority of research having been performed among women [59], and having used “non-validated assessment tools” [60]. This emphasizes the strong need for providing valid and reliable measures to assess muscularity-oriented body dissatisfaction for the Arabic-speaking population.

Through the present study, we sought to contribute to the literature especially under the Arab context, by investigating the psychometric properties of an Arabic translation of the short 9-item DMS in a sample of Arabic-speaking Lebanese university students of both genders. As mentioned above, we chose the DMS-9 because of its better conceptual clarity and good psychometric qualities [43]. Besides, this version offers potential advantages of reducing the administration time, burden, and costs. We hypothesized that the Arabic version of the DMS-9 would show good internal consistency and retain the parent two-factor structure. We also expected that the Arabic DMS’s convergent validity would be established by demonstrating theoretically coherent patterns of correlations with muscle bias internalization, muscle dysmorphic disorder, body appreciation, and disordered eating symptoms.

## Methods

### Participants

University students (N = 402; 55.2% females) from multiple universities in Lebanon filled the survey. Participants had a mean age of 24.46 years (SD = 6.60), ranging from 18 to 60 years and had a mean self-reported body mass index (BMI) 23.68 kg/m^2^ (*SD* = 4.12), ranging from 14.52 to 50.78 kg/m^2^. Most participants had a university level of education (88.8%).

Other sample characteristics are displayed in Table 1.

**Table 1:** Sociodemographic characteristics of the participants

Variable	First split-half subsample (*n* = 201)	Second split-half subsample *(n* = 201)	χ^2^ / t	*p*
Gender			0.362	0.547
Male	87 (48.3%)	93 (51.7%)		
Female	114 (51.4%)	108 (48.6%)		
Marital status			1.770	0.183
Single	162 (48.5%)	172 (51.5%)		
Married	39 (57.4%)	29 (42.6%)		
Education			0.225	0.635
Secondary or less	24 (53.3%)	21 (46.7%)		
University	177 (49.6%)	180 (50.4%)		
	**Mean ± SD**	**Mean ± SD**		
Age (in years)	25.02 ± 6.83	23.90 ± 6.33	1.711	0.088
Household crowding index	1.14 ± 0.52	1.13 ± 0.48	0.114	0.909
Physical activity	25.91 ± 18.90	25.23 ± 20.44	0.345	0.731

### Study design

Our sample was chosen using the snowball technique; a soft copy of the questionnaire was created using google forms software, and an online approach was conceived to proceed with the data collection (December 2022 and January 2023). The study’s main aims and goals, in addition to instructions for filling the questionnaire, were conveyed online for the participants, prior to their participation. Later, initial participants were asked to recruit other participants they know, preferably as diverse as possible regarding place of habitat within the Lebanese governorates. The questionnaire was anonymous and took between 15 and 20 min to complete. There were no credits received for participation. Inclusion criteria for participation included being of a resident and citizen of Lebanon of adult age. The “remove duplicates” option in excel ensured that the same answers were not submitted more than once. After providing digital informed consent, participants were asked to complete the instruments described below, which were presented in a pre-randomized order to control for order effects.

### Measures

#### Drive for muscularity scale (DMS-9)

The short form of the DMS-9 scale (9 items) was used in this study [43]. Participants indicate how each item reflects their own attitudes and behaviors on a 6-point Likert type scale ranging from 1 (not at all) to 6 (absolutely). Higher scores reflect greater drive for muscularity.

#### Muscle bias internalization scale (MBIS)

This scale is composed of 14 items scored on a 7-point Likert Scale (“1 = Strongly disagree to ‘7 = Strongly agree”; score range 14–68). Higher scores indicate higher levels of muscularity bias internalization [61]. This scale was recently validated in Arabic [62]. McDonald’s ω was .96 in the total sample.

#### Body appreciation scale-2 (BAS-2)

Validated in Arabic [63, 64], this 10-item instrument assesses acceptance of one’s body, respect and care for one’s body, and protection of one’s body from unrealistic beauty standards. All items were rated on a 5-point scale, ranging from 1 (*never*) to 5 (*always*) (score range 10–50) [42]. Higher scores on this scale reflect greater body appreciation. McDonald’s ω was 0.97 in the total sample.

#### Eating attitudes test-7 (EAT-7)

Participants were asked to complete the EAT-7, which has recently been validated in Arabic [65]. This 7-item scale measures symptoms and concerns characteristic of eating disorders. All items were rated on a 6-point scale, ranging from 0 (*never*) to 3 (*always*) (score range 0–21). Higher total scores reflect greater disordered eating attitudes. In the present study, McDonald’s ω was 0.80 in the total sample.

#### Muscle dysmorphic disorder inventory (Ar-MDDI)

Validated in the Arabic language [66], this scale is composed of 13 items, scored on a five-point Likert-type scale (0 = never to 4 = always) (score range 0–42) [67]. In the present study, McDonald’s ω was 0.88 in the total sample.

#### Demographics

Participants were asked to provide their demographic details consisting of age, gender, marital status, highest education level, self-reported height and weight to calculate the BMI, household crowding index (calculated by dividing the number of persons by that of the rooms in the house; [68]) and physical activity (calculated by multiplying the exercise strength by intensity by duration [69]).

#### Translation procedure

The DMS scale was translated to the official Arabic language, which is written and spoken across the Middle East and North Africa (MENA). The translation was performed with the purpose of achieving semantic equivalence between measures in their original and Arabic versions following international norms and recommendations [70]. To this end, the forward-backward translation approach was used. The English version was translated to Arabic by a Lebanese translator who was completely unrelated to the study. Afterwards, a Lebanese psychologist with a full working proficiency in English, translated the Arabic version back to English. The translation team ensured that any literal and/or specific translation was balanced. The initial and translated English versions were compared to detect/eliminate any inconsistencies and guarantee the accuracy of the translation by a committee of experts composed of the research team and the two translators [71]. An adaptation of the measure to the Arab context was performed, and sought to determine any misunderstanding of the items wording as well as the ease of items interpretation; and, therefore, ensure the conceptual equivalence of the original and Arabic scales in both contexts [72]. After the translation and adaptation of the scale, a pilot study was done on 20 participants to ensure all questions were well understood; no changes were applied after the pilot study.

### Analytic strategy

#### Data treatment

There were no missing responses in the dataset. To examine the factor structure of the DMS, we tested the original models proposed by McCreary et al. [22] (i.e. one- and two-factor structure in males and one-factor structure in females of the DMS-15), if divergent, we aimed at applying the EFA-to-CFA strategy of the DMS-9 [73]. To ensure adequate sample sizes for both EFA and CFA (i.e., n = 201 for EFA and CFA), we split the main sample using an SPSS computer-generated random technique; sample characteristics of the two split-halves are reported in Table 1. No significant differences were seen between the two subsamples in terms of all characteristics.

#### Exploratory factor analysis

A minimum of ten participants per scale item (i.e. 90 participants in our case) was needed to perform the EFA according to Comrey and Lee [74]. EFA was conducted via the FACTOR program using a principal-axis EFA with the first split-half subsample [75, 76]. We verified all requirements related to item-communality [77], average item correlations, and item-total correlations [78]. The Kaiser-Meyer-Olkin (KMO) measure of sampling adequacy (which should ideally be ≥ 0.80) and Bartlett’s test of sphericity (which should be significant) ensured the adequacy of our sample [79]. A preliminary analysis of the items was conducted using the Measure of Sampling Adequacy (MSA) at the item level [80], and (b) the Anti-Image Correlation (CAI) [81]. The MSA is a standardized index ranging from 0 to 1, with values below 0.50 considered unacceptable and leading to item elimination [80]. On the other hand, the Expected Residual correlation direct Change (EREC) index was used to assess the residual correlation between two items after removing the influence of all definable common factors in the dataset, hence, they should all be approximately 0. Item pairs with high-shared correlation are referred to as doublets. It is recommended to especially remove items that appear repeatedly in different doublets [82]. The procedure for determining the number of factors to extract was parallel analysis (PA; [83] using the polychoric correlation matrix since we had ordinal data. Weighted Root Mean Square Residual (WRMR) was also calculated to assess the model fit (values < 1 have been recommended to represent good fit; [84]. Item retention was based on the recommendation that items with “fair” loadings and above (i.e., ≥ 0.33).

#### Confirmatory factor analysis

CFA was conducted via the SPSS AMOS v.29 software using the maximum Likelihood estimation. The minimum sample size to conduct a CFA ranges from 3 to 20 times the number of the scale’s variables [85]. Therefore, we assumed a minimum sample of 27–180 participants needed to have enough statistical power, which was fulfilled in our second subsample. The absence of multicollinearity was verified through tolerance values > 0.2 and variance inflation factor (VIF) values < 5. Multivariate normality was not verified at first (critical ratio > 5); therefore we performed non-parametric bootstrapping procedure (only option available in AMOS). Following the guidelines in Hu and Benlter [86], the following model fit indicators were used, the normed model chi-square χ²/df (values ≤ 3 indicate good fit), the Comparative Fit Index (CFI; values close to or greater than 0.95 = good fit), the Tucker-Lewis index (TLI; values close to or greater than 0.95 = good fit), and Standardized Root Mean Square Residual (SRMR; values close to or less than 0.05 = good fit, and values between 0.06 and 0.10 = acceptable fit), the Steiger-Lind root mean square error of approximation (RMSEA) (values ≤ 0.08 reflect good fit). However, these cut-off values should not be interpreted rigidly (Heene, Hilbert, Draxler, Ziegler, & Bühner, 2011; Perry, Nicholls, Clough, & Crust, 2015) as values 0.08 to 0.10 for RMSEA can indicate acceptable but mediocre fit to the data (Hooper, Couglan, & Mullen, 2008; MacCallum, Browne, & Sugawara, 1996).

#### Gender invariance

To examine gender invariance of DMS-9 scores, we conducted multi-group CFA [87] using SPSS AMOS v.29 software on the second split-half subsample. Measurement invariance was assessed at the configural, metric, and scalar levels [88]. Proof of invariance was estimated if ΔCFI ≤ 0.010 and ΔRMSEA ≤ 0.015 or ΔSRMR ≤ 0.010 [87, 89].

#### Reliability analyses and concurrent validity

Composite reliability in both subsamples was assessed using McDonald’s ω, with values greater than 0.70 reflecting adequate composite reliability [90]. To assess convergent and criterion-related validity, we examined bivariate correlations between DMS scores and all scales included in the survey using the total sample. All scores had normal distribution, as identified by skewness and kurtosis values varying between − 1 and + 1 [91]; therefore, Student t test was used to compare two means and Pearson correlation test was used to correlate two scores. Based on [92], values ≤ 0.10 were considered weak, ~ 0.30 were considered moderate, and ~ 0.50 were considered strong correlations.

## Results

### Testing of the original DMS scale structure

The fit indices of the one- and two-factor structure of the DMS-15 conducted in males and one-factor structure in females did not show appropriate fit (Table 2).

**Table 2:** Standardized Estimates of Factor Loadings from the Confirmatory Factor Analysis (CFA) in the Second Split-Half Subsample

Group	SRMR	χ²/df	TLI	CFI	RMSEA [90% CI]
Males 1 factor	0.124	871.15/77 = 11.31	0.558	0.626	0.240 [0.226, 0.255]
Males 2 factors	0.131	467.59/76 = 6.15	0.779	0.815	0.170 [0.155, 0.185]
Females 1 factor	0.103	842.35/77 = 10.94	0.669	0.720	0.212 [0.199, 0.225]

*Note.* CFI = Comparative fit index; RMSEA = Steiger-Lind root mean square error of approximation; SRMR = Standardised root mean square residual

Consequently, we decided to conduct the EFA-CFA strategy to the DMS-9 items.

#### Exploratory factor analysis

Bartlett’s test of sphericity, χ^2^(36) = 1941.4, *p* < .001, and KMO (0.865) indicated that the DMS items had adequate common variance for factor analysis. None of the items had an MSA value < 0.5 and none of the items appeared repeatedly in different doublets; therefore, all items were kept in the analysis. The results of the EFA revealed two factors, which explained 81.44% of the common variance (item-factor loadings ≥ 0.62). The WRMR value was also adequate (= 0.077; 95% CI 0.050-0.101), indicating good fit of the model.

### Confirmatory factor analyses

The fit indices of the two-factor model of the DMS-9 scale [43] showed good results as follows: χ2/df = 67.73/26 = 2.61, TLI = 0.963, CFI = 0.973, SRMR = 0.068 and RMSEA = 0.090 [90% CI 0.064-0.116]. The standardized loading factors of the DMS scale are summarized in Table 3.

**Table 3:** Items of the Drive for Muscularity Scale in English and Factor Loadings Derived from the Exploratory Factor Analyses (EFA) in the first split-half subsample and Standardized Estimates of Factor Loadings from the Confirmatory Factor Analysis (CFA) in the Second Split-Half Subsample

	EFA		CFA
Item	Factor 1	Factor 2	Total
1. I wish that I were more muscular.	− 0.10	0.89	0.74
2. I use protein or energy supplements	0.97	− 0.06	0.93
3. I drink protein shakes to increase weight	0.97	− 0.07	0.98
4. I try to consume as many calories as I can in a day	0.81	0.05	0.72
5. I feel guilty if I miss a weight training session	0.90	− 0.01	0.77
6. I think about taking anabolic steroids	0.70	0.19	0.71
7. I think that my arms are not muscular enough	0.06	0.92	0.92
8. I think that my chest is not muscular enough	0.11	0.89	0.90
9. I think that my legs are not muscular enough	− 0.02	0.95	0.92

Note: Factor 1 = Muscularity Behaviors; Factor 2 = Muscularity body Dissatisfaction

### Internal consistency

McDonald’s omega values were 0.90 for Factor 1 (Muscularity Behaviors) and 0.92 for Factor 2 (Muscularity body Dissatisfaction).

### Measurement invariance

The fit indices in Table 4 suggest measurement invariance of the DMS-9 scores across genders. Higher mean Muscularity behaviors and Muscularity body dissatisfaction scores were significantly found in males compared to females (12.21 ± 5.43 vs. 9.73 ± 5.08; p = .001 and 11.69 ± 6.50 vs. 9.54 ± 5.80; p = .015) respectively.

**Table 4:** Measurement Invariance Across Gender in the Second Split-Half Subsample

Model	χ²	*df*	CFI	RMSEA	SRMR	Model Comparison	Δχ²	ΔCFI	ΔRMSEA	ΔSRMR	Δ*df*	*p*
Configural	127.77	56	.954	.080	.151							
Metric	148.81	63	.945	.083	.125	Configural vs metric	21.04	.009	.003	.026	7	.003
Scalar	164.53	70	.940	.082	.125	Metric vs scalar	15.72	.005	.001	< .001	7	.027

*Note.* CFI = Comparative fit index; RMSEA = Steiger-Lind root mean square error of approximation; SRMR = Standardised root mean square residual

### Convergent and divergent validity

Higher Muscularity behaviors and Muscularity body dissatisfaction scores were significantly associated with more muscle bias internalization, muscle dysmorphic disorder and inappropriate eating attitudes, and lower body appreciation (Table 5).

**Table 5:** Correlation of continuous variables

	1	2	3	4	5	6
1. DMS-9 Muscularity behaviors	1					
2. DMS-9 Muscularity body dissatisfaction	0.54***	1				
3. Muscle bias internalization	0.52***	0.52***	1			
4. Muscle dysmorphic disorder	0.55***	0.65***	0.51***	1		
5. Body appreciation	− 0.19***	− 0.24***	− 0.30***	− 0.38***	1	
6. Eating attitude test	0.17**	0.17**	0.14**	0.24***	− 0.004	1

MDDI = Muscle Dysmorphic Disorder Inventory; DMS-9 = Drive for masculinity scale-9

## Discussion

The present study was conducted with the aim of making available an Arabic psychometrically sound measure to assess drive for muscularity, i.e. the short 9-item DMS. The Arabic version was found to have excellent psychometric properties in terms of factorial structure, internal consistency, gender invariance, and convergent/divergent validity. These findings preliminarily suggest that the Arabic DMS is a simple, easy to use, and economic self-report scale for the reliable and valid assessment of drive for muscularity among Arabic-speaking community people.

Using an EFA-to-CFA strategy as recommended in the literature [73], we found that the original two-factor model of the DMS proposed in the parent study [22] was adequately replicated in our sample; suggesting that this structure is appropriate for the Arabic-speaking population. In agreement with our findings, most of the linguistic validations of the DMS confirmed the originally proposed two-factor structure, including the Spanish [20], German [26], Malay [32], and Mexican versions [24]. Nevertheless, findings on factorial validity of the DMS seem to be conflicting. Some studies, indeed, failed to support this model; and rather recommended the use of the general scale (e.g., [31]). Other researchers attempted to test a hypothesized three-factor model (e.g., [24, 27]). Other translation studies demonstrated the good internal consistency of the DMS but omitted to explore its factor structure (e.g., Swedish [93] Icelandic [94], French [95]). Finally, and as previously mentioned, evidence for the two-factor model mainly derived from men samples [39]; while those involving women did not confirm this model (e.g., [22, 40, 41]).

The two DMS-9 factor scores showed very good McDonald’s omega values (ω > 0.8), higher than the 0.70 threshold value of good internal consistency suggested by previous researchers [96, 97], thus suggesting that the present Arabic version of the DMS appears to offer a reliable measure of drive for muscularity manifestations. These findings are consistent with the original validation of the short 9-item DMS, which revealed a Cronbach’s alphas for the Muscularity Behaviors and the Muscularity Body Dissatisfaction subscales of 0.88 and 0.87, respectively [43]. Overall, the present results corroborate previous evidence that the DMS is consistently reliable [39]. Beyond reliability, our study is among the first to examine measurement invariance of the DMS across men and women, in a relatively proportionate sample of adults according to gender (51.4% women in the first sample and 48.6% women in the second sample). Despite evidence showing that drive for muscularity could manifest among females [98–100], gender-related aspects with regard to this entity have long been neglected. This has led Kling et al. [39] to call in their systematic review for future studies extending investigations of the construct beyond men samples, and examining cross-gender invariance of the DMS. Findings showed that gender invariance was achieved at the configural, metric, and scalar levels. These findings suggest that items are interpreted by, and applicable to men and women in the same manner; thus allowing for valid gender comparisons in future research. Additional studies are warranted to replicate and confirm these findings.

Finally, DMS-9 scores correlated in the expected way with other study variables, providing support for the convergent and divergent validity of the Arabic version of the scale. Specifically, we found that greater drive for muscularity attitudes and behaviors significantly correlated with more severe muscle dysmorphic symptoms, inappropriate eating attitudes, muscle bias internalization, and lower body appreciation. These results align with previous evidence. Similar evidence for validity of the DMS-9 through the same patterns of correlations with these variables has previously been reported in other validation studies (e.g., disordered eating attitudes/behaviors [1, 20, 31], body image dissatisfaction [23, 42, 43], muscle dysmorphic disorder [33, 101]). These data further highlight the clinical relevance of the drive for muscularity construct, and suggests that efforts to help people address this issue may be beneficial for their health and well-being [5].

### Limitations

Some limitations should be acknowledged. First, our data were collected following convenience sampling and a web-based survey; which might limit the generalization of our conclusions. Information is present in all cross-sectional studies. We could not verify if a participant took the survey more than once. Moreover, linguistic invariance was not studied; the scale should be tested for being valid and reliable to use in other Arab countries due to the complexity of the Arabic language and its vernacular forms. More validation studies still need to confirm the robustness of the Arabic DMS-9 in specific groups (such as Arabic-speaking athletes, bodybuilder and sexual minority individuals). In addition, future cross-national validations in samples from different Arab countries are required to provide support to the cross-cultural validity of the scale. Finally, other important psychometric properties have not been addressed in this paper (e.g., test-retest reliability, construct validity) and should be verified in future research.

## Conclusion

Through this study, we provide a brief, valid, economic and useful tool to evaluate drive for muscularity in Arabic-speaking men and women. Making the Arabic DMS-9 available will hopefully benefit the scientific community working in Arab settings, promote local and international research in this area, and offer descriptive data on how drive for muscularity may interfere with health indicators in the general Arab population.

## Data Availability

All data generated or analyzed during this study are not publicly available due the restrictions from the ethics committee. Data can be shared upon a reasonable request to the corresponding author.
